# N-GlyDE: a two-stage N-linked glycosylation site prediction incorporating gapped dipeptides and pattern-based encoding

**DOI:** 10.1038/s41598-019-52341-z

**Published:** 2019-11-04

**Authors:** Thejkiran Pitti, Ching-Tai Chen, Hsin-Nan Lin, Wai-Kok Choong, Wen-Lian Hsu, Ting-Yi Sung

**Affiliations:** 1 Institute of Information Science, Academia Sinica, Taipei, 11529 Taiwan; 2 Institute of Bioinformatics and Structural Biology, National Tsing Hua University, Hsinchu, 30013 Taiwan; 3 Bioinformatics Program, Taiwan International Graduate Program, Institute of Information Science, Academia Sinica, Taipei, 11529 Taiwan

**Keywords:** Sequence annotation, Machine learning

## Abstract

N-linked glycosylation is one of the predominant post-translational modifications involved in a number of biological functions. Since experimental characterization of glycosites is challenging, glycosite prediction is crucial. Several predictors have been made available and report high performance. Most of them evaluate their performance at every asparagine in protein sequences, not confined to asparagine in the N-X-S/T sequon. In this paper, we present N-GlyDE, a two-stage prediction tool trained on rigorously-constructed non-redundant datasets to predict N-linked glycosites in the human proteome. The first stage uses a protein similarity voting algorithm trained  on both glycoproteins and non-glycoproteins to predict a score for a protein to improve glycosite prediction. The second stage uses a support vector machine to predict N-linked glycosites by utilizing features of gapped dipeptides, pattern-based predicted surface accessibility, and predicted secondary structure. N-GlyDE’s final predictions are derived from a weight adjustment of the second-stage prediction results based on the first-stage prediction score. Evaluated on N-X-S/T sequons of an independent dataset comprised of 53 glycoproteins and 33 non-glycoproteins, N-GlyDE achieves an accuracy and MCC of 0.740 and 0.499, respectively, outperforming the compared tools. The N-GlyDE web server is available at http://bioapp.iis.sinica.edu.tw/N-GlyDE/.

## Introduction

Protein glycosylation is a highly complex post-translational modification (PTM) that influences a variety of biological processes as protein folding, signaling, trafficking, cell-cell interactions, and immune response^1–3^. In marketed therapeutic proteins, more than one-third of approved biopharmaceuticals belong to glycoproteins^4^. More than 50% of all polypeptides are covalently modified by the addition of structurally diverse oligosaccharides to specific functional groups^5^. Based on the nature of the chemical linkage between the specific acceptor amino acid and the glycan, glycosylation can be classified into four major categories: N-linked and O-linked glycosylation, C-mannosylation, and glycosylphosphatidylinositol (GPI) anchors^6^. Among these, N-linked glycosylation is the most abundant type. Therefore, in this paper we particularly study N-linked glycosylation on the human proteome.

In N-linked glycosylation, the glycan is attached to the amide nitrogen of asparagine (N). More specifically, N-linked glycosylation predominantly occurs in N-X-S/T (S: serine, T: threonine) sequons, and in some rare cases N-X-C (C: cysteine), where X can be any amino acid except proline^7^. Though the presence of a sequon is necessary, it alone is not a sufficient criterion for glycosylation as one-third of the asparagine in the sequons are not found to be glycosylated^8^. Identifying the substrates and their corresponding glycosylation sites is essential to understand the mechanism of N-linked glycosylation^9,10^. However, experimental characterization of N-linked glycosites in glycoproteins is challenging because it is technically demanding, expensive, and time-consuming^11^. Therefore, there is a pressing need to develop methods to predict glycan occupancy at the N-X-S/T sequons of proteins.

There are two types of prediction methods, depending on the types of features used in machine learning, namely, sequence-based predictors which use protein sequence information alone, and structure-based predictors which use experimentally solved structure information in addition to sequence information. Currently, several sequence-based predictors have been made available. For example, NetNGlyc uses neural networks for prediction^12^. EnsembleGly^13^ uses an ensemble of support vector machine (SVM) classifiers developed on 254 N-linked glycosites and 1469 non-N-linked glycosites. GPP (Glycosylation Prediction Program)^14^ uses the random forest algorithm developed on 261 N-linked glycosites and 3247 non-N-linked glycosites for prediction. Both EnsembleGly and GPP use a relatively smaller dataset of annotated glycoproteins for model development. More recently, GlycoEP^15^ uses SVMs for prediction, in which the authors extract experimentally-determined eukaryotic glycoproteins from Swiss-Prot and obtain 1797 N-linked glycoproteins to develop the predictor on two datasets, standard and advanced, having different levels of redundancy reduction in protein sequences. GlycoMine^16^ uses the random forest approach, in which 416 experimentally verified glycosites in human proteins are considered positive sites and asparagines in the sequons from proteins other than experimentally-validated glycoproteins are considered negative sites. SPRINT-Gly^17^ uses a deep neural network approach on 2369 human proteins and 2096 mouse proteins for N-linked glycosite prediction. In these sequence-based predictors, various features are used, including amino acid composition, AAIndex, position-specific scoring matrix (PSSM), predicted secondary structure, predicted surface accessibility, and predicted disordered region. Fewer structure-based predictors have been made available due to the limited number of experimentally solved structures. For example, NGlycPred^18^ uses random forests for prediction, in which structural, sequence, and pattern properties are utilized for model development. GlycoMine^struct^ ^19^ also uses random forests for model development with an additional two-stage strategy to select features among sequence- and structure-based features.

Since the number of solved structures deposited in Protein Data Bank^20^ is still limited and since query proteins may not have solved structures, we are interested in developing a sequence-based prediction method. Although the above-mentioned sequence-based predictors all report high accuracy, their performance, except that of NetNGlyc, were evaluated on each N in the protein, which is frequently not part of the N-X-S/T sequon. Performance evaluation without being confined to the N-X-S/T sequon may result in performance overestimation, and a naive predictor that predicts each N in the N-X-S/T sequon as a glycosylation site achieves comparable performance. Furthermore, we also observe that the definitions of positive and negative cases in the datasets for machine learning and testing are also crucial for performance evaluation. Clearly, experimentally validated sequons are considered positive cases. However, for an N-X-S/T sequon in experimentally validated glycoproteins annotated as glycosite by sequence analysis in UniProt^21^, we cannot be certain about its experiment evidence. It may be or may not be a positive case, and thus should be excluded from performance evaluation.

Therefore, in this paper we propose N-GlyDE, a two-stage prediction method that replaces direct glycosite prediction for use in N-linked glycosite prediction on human proteins, as therapeutics based on glycoproteins is gaining more attention. The first stage uses a protein similarity voting algorithm based on both glycoproteins and non-glycoproteins to provide a prediction score for adjusting glycosite prediction results. The second stage uses an SVM trained on glycoproteins only to predict for each N-X-S/T sequon in the query protein whether it is a glycosylation site. N-GlyDE then integrates the results of the first-stage prediction for weight adjustment of the second-stage prediction results to yield the final prediction for each sequon. As features the SVM model uses gapped dipeptides (GD), solvent accessibility (SA), and secondary structure (SS). Notably, the latter two types of features are encoded using a pattern-based approach to reduce the feature dimensions, as the training dataset contains only of a limited number of glycoproteins. The performance of N-GlyDE has been evaluated on an independent dataset in comparison with existing tools, including GlycoMine, GlycoEP, and NetNGlyc.

## Results and Discussion

### Overview of N-GlyDE

**Figure 1:**
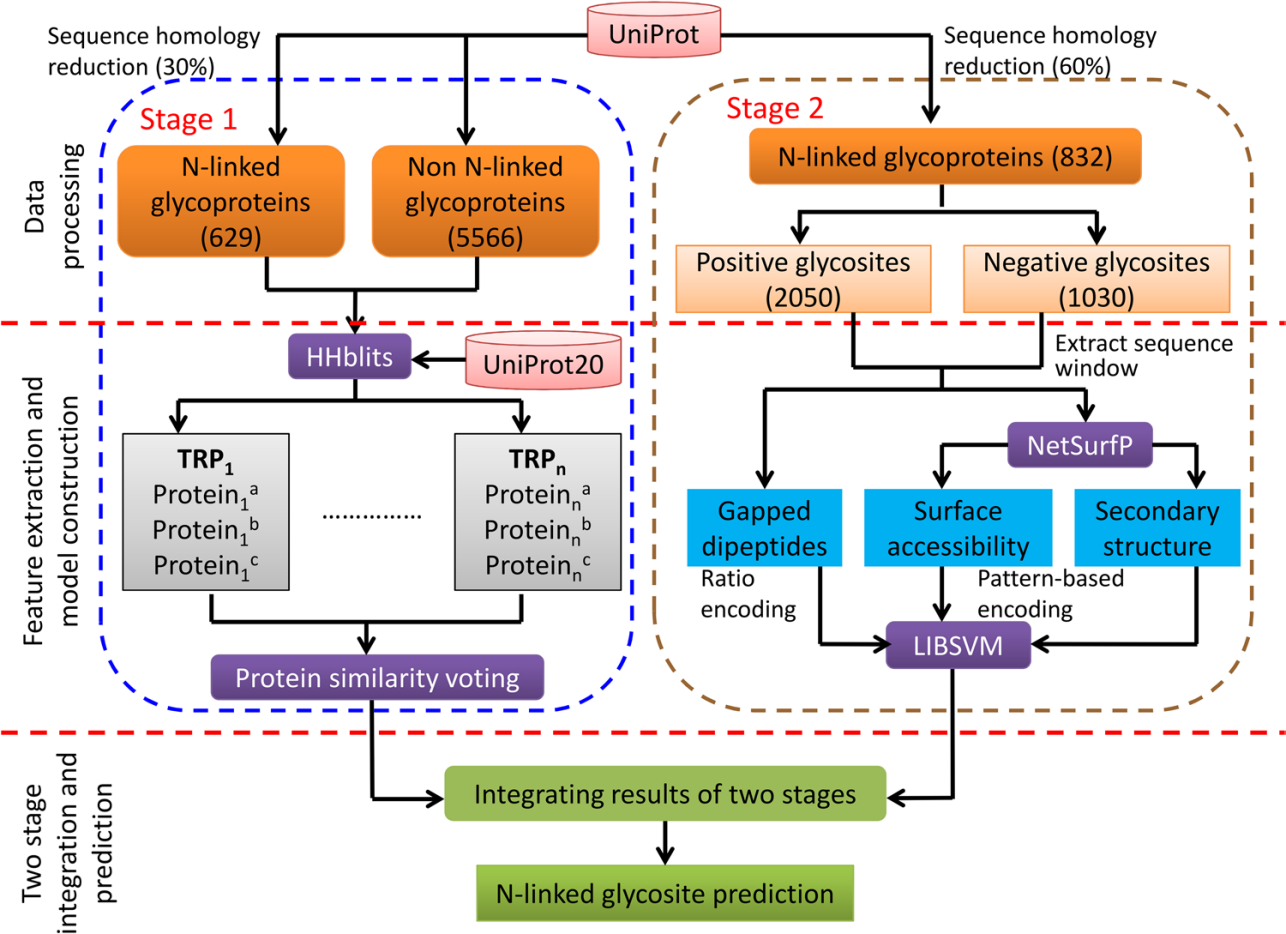
Schematic framework of N-GlyDE.

To predict N-linked glycosites, we propose N-GlyDE, a two-stage prediction method.. In the first stage, we describe an algorithm that generates a score for each protein that is integrated with the second-stage results to improve direct (one-stage) glycosite prediction. The second stage uses SVM to predict the possibility of each N-X-S/T sequon in the query protein to be glycosylated. The output of the second-stage predictor is weighted by the score from the first-stage predictor through a heuristic approach based on score thresholds to obtain the final prediction output. The overall framework of N-GlyDE is illustrated in Fig. 1.

### Rationalization of score thresholds for weight adjustment to obtain final prediction and cross-validation performance of SVM prediction

**Figure 2:**
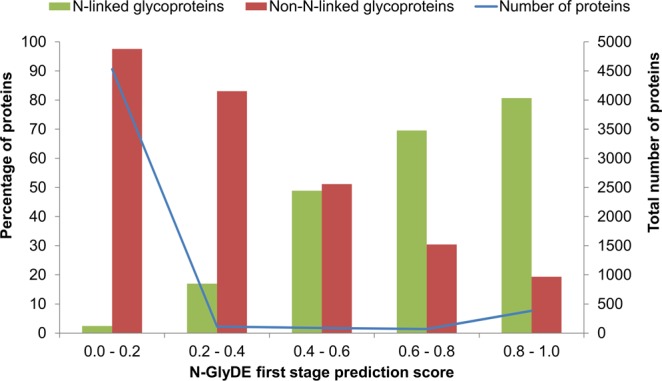
N-GlyDE first-stage prediction on 6195 proteins. The green and red bars indicate the percentage of N-linked glycoproteins and non-N-linked glycoproteins predicted at each interval. The blue line represents the total number of proteins observed across each interval.

Ten-fold cross validation was performed on both first- and second-stage datasets (Supplementary File 1: Worksheets W1 and W2) for rationalization of score thresholds for weight adjustment and for performance evaluation of SVM prediction, respectively. We obtained the prediction score of each protein in the entire dataset (6195 proteins) of glycoproteins and non-glycoproteins based on the cross-validated first-stage prediction (see the “Methods” section). Prediction scores were divided into five intervals; the complete set of proteins were grouped by prediction scores accordingly. The percentage of N-linked glycoproteins with prediction score at each interval, denoted as *p*_*i*_, defined as the number of glycoproteins divided by the total number of proteins with prediction score at this interval, is shown to reveal a positive correlation with prediction score, whereas the percentage of non-N-linked glycoproteins (i.e., 1 - *p*_*i*_) and prediction score reveals a negative correlation, as shown in Fig. 2. It is also observed that 80.7% (309 out of 383) proteins above the prediction score of 0.8 are N-linked glycoproteins and 97.2% (4511 out of 4641) proteins below the prediction score of 0.4 are non-N-linked glycoproteins. The scores of 0.4 and 0.8 are thus used as thresholds for weight adjustment of second-stage glycosite prediction results to determine the final prediction output on the independent dataset.

Based on ten-fold cross validation, the second-stage prediction (see the “Methods” section) on the 3080 sequons in the second-stage dataset achieved an MCC (Matthews correlation coefficient), accuracy, precision, sensitivity, and specificity (see the “Methods” section) of 0.413, 0.733, 0.811, 0.781, and 0.639, respectively.

### Performance evaluation and comparison with existing predictors on the independent dataset

Using N-GlyDE’s second-stage prediction alone on the independent dataset (Supplementary File 1: Worksheet W3) yielded an MCC, accuracy, precision, sensitivity, and specificity of 0.353, 0.655, 0.526, 0.784, and 0.578, respectively. In particular, the second stage of N-GlyDE predicted 131 true positives (TPs) and 36 false negatives (FNs) of 167 glycosites, and 162 true negatives (TNs) and 118 false positives (FPs) of 280 non-glycosites. In contrast, using N-GlyDE, i.e., the integration of two stages (see the “Methods” section), led to an MCC, accuracy, precision, sensitivity, and specificity of 0.499, 0.740, 0.613, 0.826, and 0.689, respectively, an improvement of 14.6%, 8.5%, 8.7%, 4.2%, and 11.1% over those of second-stage prediction alone. This improvement is attributed to the integration of the two stages by weight adjustment, rendering seven previous FNs to TPs and 31 previous FPs to TNs, as shown in Supplementary File 2: Fig. S1. The results show that integrating the result of the first stage for weight adjustment of glycosite prediction yields better prediction.

**Figure 3:**
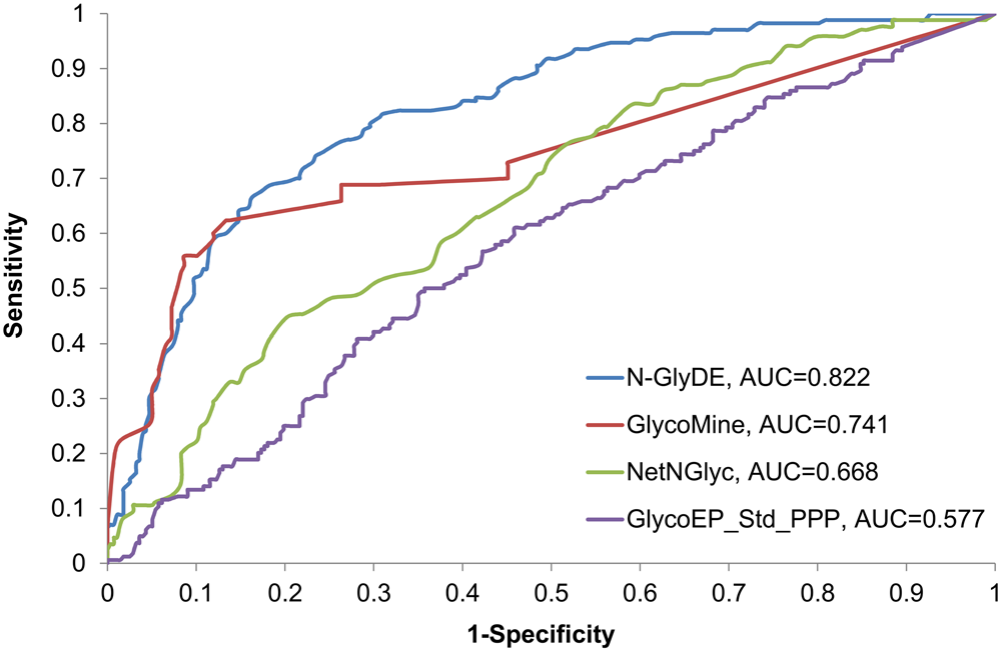
ROC curves of N-GlyDE, GlycoMine, NetNGlyc, and GlycoEP_Std_PPP on the independent dataset. For each predictor, the area under the ROC curve is calculated.

Next, we further compared the performance of N-GlyDE with the publicly-available predictors NetNGlyc, GlycoMine, and GlycoEP on the independent dataset. In particular for GlycoEP, all of the available four models of the standard predictor and two models of the advanced predictor were tested. Among these six GlycoEP models, the Std_PPP model, which uses the PSSM profile of patterns seen in the standard predictor, achieved the best MCC; its performance is reported for comparison. The performance of these predictors is shown in Table 1. N-GlyDE achieves the highest accuracy and MCC, while GlycoMine achieves the highest precision and specificity; NetNGlyc achieves the best sensitivity. We also note that the MCC value of all compared methods, including N-GlyDE, is relatively low in comparison to those of other tools reported in the literature because of the fundamental difference in the assessment of N-linked glycosylation sites. In this study, the performance of glycosite prediction is evaluated on the N-X-S/T motif, whereas most existing predictors evaluate the performance on every asparagine, not necessarily in the motif. Asparagine occurring outside an N-X-S/T motif usually does not contribute to N-linked glycosylation. A large number of asparagine outside the N-X-S/T motif can be easily predicted as non-glycosites and regarded as true negatives, thereby rendering a higher MCC. The ROC (receiver operating characteristic) curves of these predictors are shown in Fig. 3; N-GlyDE has the highest area under the curve (AUC). In summary, N-GlyDE outperforms the other three methods in MCC and AUC and achieves the second best precision, sensitivity, and specificity.

**Table 1:** Prediction performance of different predictors on the independent dataset.

Predictors	Accuracy	Precision	Sensitivity	Specificity	MCC
N-GlyDE	**0.740**	0.613	0.826	0.689	**0.499**
GlycoMine	0.725	**0.616**	0.700	**0.739**	0.430
NetNGlyc	0.572	0.460	**0.844**	0.411	0.265
GlycoEP_Std_PPP	0.574	0.437	0.512	0.610	0.119

### Gapped dipeptides as useful features

N-GlyDE’s second-stage predictor, i.e., an SVM glycosite predictor, generates predictions using three types of features: gapped dipeptides (GD), surface accessibility (SA), and secondary structure (SS) (see the “Methods” section). We evaluated the contribution of different features by comparing the MCC of the predictor on the second-stage dataset. The MCC when using different feature sets, i.e., SA + SS, GD alone, GD + SS, GD + SA, and GD + SA + SS, are 0.200, 0.360 0.376, 0.378, and 0.413, respectively. The ROC curves and AUC when using the above feature sets are shown in Supplementary File 2: Fig. S2. Notably, using GD alone as features yields an MCC of 0.360, better than using both SA and SS as features. Moreover, using GD + SS or GD + SA as features improves the MCC of using GD alone only slightly, by at most 0.018. Using GD + SA + SS as features achieves a better MCC than using GD alone by 0.053. Similar to MCC, AUC reveals the same trend among different feature sets; using GD alone as features yields a better AUC than using SA + SS. The MCC and AUC results show that gapped dipeptides are crucial and contribute the largest improvement on prediction performance in comparison with other features.

We further examined the gapped dipeptides with the top and bottom 10 GDR (gapped dipeptide ratio) (see the “Methods” section) derived from 832 glycoproteins of the second-stage dataset, which are considered discriminative and specifically positive-oriented gapped dipeptides and negative-oriented gapped dipeptides, as listed in Table 2. Frequent occurrences of amino acids such as tryptophan (W) and tyrosine (Y) (6 out of 10) are exclusively observed only in positive-oriented gapped dipeptides, as shown in Table 2. Both of these amino acids are nonpolar in nature and have an aromatic ring as a part of their side chain. The presence of a hydroxyl group on the phenyl ring in tyrosine and an indole ring with nitrogen in tryptophan engage these amino acids in hydrogen bond formation^22,23^. Free N-glycans of both the high-mannose and complex types having a binding affinity for aromatic amino acid residues were evident from the work of Yamaguchi *et al*.^24^. Based on our observation, we suggest that the presence of amino acids W and Y neighboring the sequon contributes to N-linked glycosylation.

**Table 2:** Discriminative gapped dipeptides with high and low GDR.

Positive-oriented gapped dipeptides	Top 10 GDR	Negative-oriented gapped dipeptides	Bottom 10 GDR
W5**N**	2.401	**N**2P	0.064
**N**2M	2.198	C4**N**	0.542
W11**N**	1.96	**N**4K	0.569
Y7**N**	1.96	K11**N**	0.574
**N**0Y	1.941	**N**7M	0.589
L0**N**	1.923	N0**N**	0.595
Y0**N**	1.884	M9**N**	0.612
**N**10K	1.794	**N**8P	0.618
W0**N**	1.794	K6**N**	0.63
H6**N**	1.714	**N**0K	0.643

Underlined and boldface asparagine corresponds to the sequon and is located at the center of a sequence window.

### Case studies

**Figure 4:**
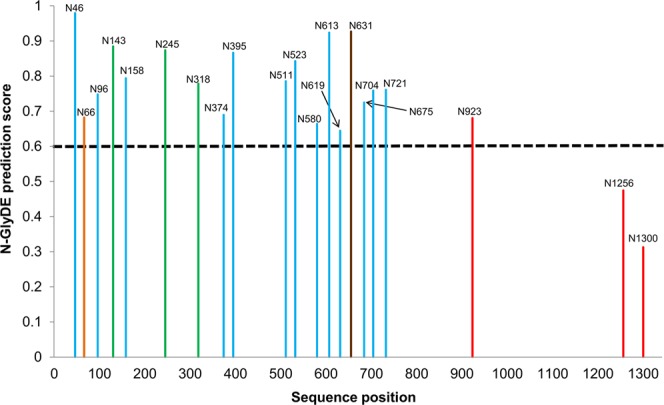
N-GlyDE prediction results of human VEGFR2, where sites with a prediction score above 0.6 (shown by the dotted line) are predicted as glycosites. Green bars represent the three glycosites with experimental evidence in UniProt. Glycosites annotated by sequence analysis in UniProt were recently validated by Chandler *et al*.’s mass spectrometry (MS) experiment on extracellular domain of murine VEGFR2, which shares 86% sequence similarity with human VEGFR2. Blue bars represent the MS-validated glycosites; the brown bar (N631) represents sites undetected in the MS experiment; and the orange bar (N66) represents the sequon only in human, not in murine. Red bars represent the non-glycosites, which was not studied by Chandler *et al*. The number following ‘N’ represents the asparagine position of the sequon in the sequence.

We performed two case studies on proteins in the independent dataset. First, the vascular endothelial growth factor receptor 2 (VEGFR2_HUMAN, UniProt ID: P35968), a protein of the receptor-type tyrosine kinase (RTK) family, was investigated to demonstrate the competence of N-GlyDE. VEGFR2 plays a crucial role in vascular endothelial cell development by regulating its function, and is also important for regulating angiogenesis and lymphangiogenesis^25,26^. It contains 21 N-X-S/T sequons, where three N at positions 143, 245, and 318 are experimentally validated glycosites^27,28^, 15N are annotated in UniProt as glycosites by sequence analysis, and three N at positions 923, 1256, and 1300 so far have no evidence to undergo glycosylation. Note that only six sequons, i.e., three glycosites and three non-glycosites, were included for evaluating the performance on the independent dataset. The predicted results of the 21 sequons in VEGFR2 by N-GlyDE are shown in Fig. 4. N-GlyDE correctly predicts the three validated glycosites and two (at positions 1256 and 1300) out of three non-glycosites, achieving an accuracy of 83.3% (=5/6). In addition, N-GlyDE predicts the 15 sequons with evidence at the sequence analysis level as positives. To evaluate the accuracy of N-GlyDE’s prediction on these 15 sequons with sequence analysis evidence, we note a recent work by Chandler *et al*.^29^ that uses tandem mass spectrometry to study site-specific glycosylation of extracellular domains in murine VEGFR2, which shares 86% sequence identity with human VEGFR2, suggesting that the outcome of murine VEGFR2 is applicable to human VEGFR2. Extracellular domains of murine VEGFR2 possess 17 sequons, whereas human VEGFR2 possesses 18 sequons. The additional sequon in the human VEGFR2 is found at position 66, which has glycosylation annotation by sequence analysis, and is excluded from this case study. In Chandler *et al*.’s study, 13 out of the 14 sequons with sequence analysis evidence were confirmed as glycosites except for the sequon at position 631 of the human VEGFR2, as the corresponding peptide is undetected in the mass spectrometry experiment. N-GlyDE correctly predicts these 13 glycosites and achieves an accuracy of 94.7% (=18/19). Finally, regarding the asparagine at position 923 that is not included in Chandler *et al*.’s study, although it is considered a non-glycosite according to the annotation in UniProt, it is predicted as a glycosite by N-GlyDE. However, whether it will be glycosylated awaits experimental validation. Supplementary File 2: Table S1 lists prediction results of N-GlyDE for all the sequons in VEGFR2 along with the UniProt evidences and observations from Chandler *et al*.’s work.

Second, to demonstrate the efficacy of gapped dipeptides for glycosite prediction, fibronectin (FN, UniProt ID: P02751) was investigated. It is a large glycoprotein involved in diverse biological functions such as nerve regeneration, embryogenesis, and many adhesive functions^30^. It contains ten N-X-S/T sequons of which seven N are experimentally validated glycosites and three N at positions 1236, 1417, and 1813 so far have no evidence to undergo glycosylation as annotated in UniProt. N-GlyDE accurately predicts six out of seven validated glycosites as positives and all of the three non-glycosites as negatives, yielding an accuracy of 90%. Notably, five of the six true-positive glycosites at positions 430, 542, 877, 1244, and 2108 exhibit occurrences of some positive-oriented gapped dipeptides such as N0Y, H6N, L0N, and Y0N as shown in Supplementary File 2: Table S2. Furthermore, all of the three true negative sequons display occurrences of some negative-oriented gapped dipeptides such as N2P as shown in Supplementary File 2: Table S2. The results show the efficacy of positive-oriented and negative-oriented gapped dipeptides. Supplementary File 2: Table S2 lists the highly significant positive-oriented and negative-oriented gapped dipeptides observed in these sequons along with UniProt evidences and the N-GlyDE prediction scores.

## Conclusion

In this study, we develop N-GlyDE, a two-stage sequence-based prediction model trained on rigorous non-redundant glycoproteins and non-glycoproteins to predict N-linked glycosylation sites in human proteins. The first stage provides a prediction score for every protein based on which the glycosite prediction score of the second stage is adjusted. Performance evaluation was conducted on N-X-S/T sequons of an independent dataset, comprised of 53 glycoproteins and 33 non-glycoproteins. Only experimentally validated glycosites are considered positives. Asparagines in the sequon of a glycoprotein without any annotation and asparagines of all N-X-S/T sequons in non-glycoproteins are regarded as negatives. Compared with the GlycoMine, NetNGlyc, and GlycoEP predictors, N-GlyDE outperforms them in terms of MCC by 6.9%, 23.4%, and 38%, respectively. Out of the three types of features used in N-GlyDE’s SVM predictor, gapped dipeptides turn out to be more discriminative than secondary structure and surface accessibility features. Frequent occurrences of amino acids with aromatic side chains such as tyrosine and tryptophan seem to positively correlate the attachment of N-glycan to the asparagine of the sequon. In the future, predicted structural features, e.g., disordered regions, and physicochemical property features can be considered to further improve the predictor. When more glycoproteins are experimentally validated, deep learning can be used for prediction.

## Methods

### Datasets

Human proteins in UniProt (ver. 201608) are used as our source. To compile the positive dataset, i.e., experimentally validated N-linked glycoproteins, we searched for “glycosylation” in the PTM field and added a filter using the keyword “N-linked”. In addition, all the proteins must be “reviewed” and from “Homo sapiens” and have evidence as “experimental assertion”. A similar search strategy was used to select the negative cases, i.e., non-N-linked glycoproteins, by setting the filter to exclude N-linked glycoproteins in the PTM field with evidence of any assertion. As a result, we obtained 1068 glycoproteins and 11767 non-glycoproteins. We used an in-house script to remove glycoproteins with only a single experimentally validated sequon having proline at the center or any amino acid apart from serine or threonine at the third position. Furthermore, we also confirmed experimentally validated N-linked glycosites by checking the reported experiments performed for that position in the cited literature, in addition to their evidence codes in UniProt. After this rigorous procedure, 1011 glycoproteins remained. In order to construct non-redundant datasets, we used CD-HIT^31^ to remove protein sequences with a sequence similarity of over 30% in both positive and negative datasets, yielding 682 glycoproteins and 5599 non-glycoproteins, a total of 6281 non-redundant proteins.

For glycosite prediction, we considered only the experimentally validated sequons as positive cases. Sequons having evidence such as ‘Probable’, ‘Potential’, or ‘By sequence similarity’ were excluded from training and performance evaluation. Both glycoproteins and non-glycoproteins contributed for non-glycosites, i.e., negative cases. Because currently no technology is available to exactly identify which asparagine in the protein sequence does not undergo N-linked glycosylation, the selection of non-glycosylation sites (from N-linked glycoproteins) is difficult; thus we consider sequons without any evidence of undergoing N-linked glycosylation so far in glycoproteins as negative cases, which were used in the second-stage dataset. All the sequons in non-glycoproteins were regarded as negatives, which were combined with negative cases from glycoproteins for performance evaluation on the independent dataset.

#### Independent dataset

Since query proteins can be glycoproteins or non-glycoproteins, we developed an independent dataset containing both types of proteins. Among these 6281 non-redundant glycoproteins and non-glycoproteins, we randomly selected 86 proteins, consisting of 53 glycoproteins and 33 non-glycoproteins, as the independent dataset, where non-glycoproteins were chosen from the nucleus, cytosol, and mitochondrion since proteins from these subcellular localizations are known not to undergo N-linked glycosylation^32^. Selection of non-glycoproteins was restricted to these three subcellular localizations to ensure that the sequons from these proteins do not undergo N-linked glycosylation and remain true negatives. The 53 glycoproteins contained 167 confirmed glycosites and 64 non-glycosites, and their 112 sequons having evidence of ‘Probable’, ‘Potential’, or ‘By sequence similarity’ were omitted. The 33 non-glycoproteins contained 216 non-glycosylated sequons. In other words, 447 N-X-S/T sequons, i.e., 167 glycosites and 280 non-glycosites, in the independent dataset were used for performance evaluation.

#### First-stage dataset

Excluding the independent dataset, the remaining 629 glycoproteins and 5566 non-glycoproteins, a total of 6195 proteins collected across various subcellular localizations, were used in the first-stage training, which yields a prediction score for every protein.

#### Second-stage dataset

For glycosite prediction, we used N-X-S/T sequons in the glycoproteins to train the predictor. The 629 non-redundant glycoproteins with at most 30% sequence identity contained 1547 glycosylated sequons (glycosites) and 828 non-glycosylated sequons (non-glycosites), a total of 2375 sequons. To better develop our features and train the SVM model, we enlarged the second-stage dataset by relaxing the sequence identity threshold to 60% and obtained 832 glycoproteins, which contained 3080 sequons, including 2050 glycosylated sequons and 1030 non-glycosylated sequons. Although the redundancy reduction threshold was relaxed to 60%, we also used CD-HIT to ensure that the sequence identity between independent dataset and second-stage dataset remains at most 30%.

### N-GlyDE method

As described below, N-GlyDE is a two-stage method to predict N-linked glycosites in proteins.

### First stage of N-GlyDE

Conventional sequence similarity search tools such as PSI-BLAST^33^ can be used to easily determine whether a protein is an N-linked glycoprotein through a set of well-aligned sequence partners from existing protein databases. However, the prediction problem becomes challenging when the search is limited to a non-redundant protein sequence dataset and the sequence homology is not easily obtained. We propose a protein similarity voting algorithm to solve the problem using HHblits^34^, a homologous sequence detection method based on iterative HMM-HMM (hidden Markov model) comparison. Similar to PSI-BLAST, HHblits iteratively searches for homologies in a protein database by performing profile-profile alignments, which is implemented as a sequence-to-profile comparison by converting the vector of 20 amino acid probabilities into an alphabet, representing a typical profile column. Thus HHblits is faster than PSI-BLAST owing to the discretized design of HMM and is reported to yield 50–100% higher sensitivity in finding distant homologs^34^. Thus, we use HHblits to find homologs for each protein sequence.

Given a protein *P* from the training set, HHblits is used to search against its built-in uniprot20_2016_02 human proteins to identify top-ranked proteins (TRP). Proteins in TRP share pairwise sequence similarities of less than 20%, and each is reported with a probability denoting its similarity to protein *P*. All the proteins in TRP with probabilities below 0.5 are removed, and the remaining proteins form a set of similar proteins with respect to protein *P*, denoted as S(*P*). HHblits search is performed iteratively for each of the proteins in the first-stage training set. Given a query protein *Q* from the test set, HHblits is again used to search against the built-in uniprot20_2016_02 to construct S(*Q*) as described above. Then S(*Q*) is compared with each *S*(*P*) for all proteins *P* in the training set. If S(*Q*) and S(*P*) have at least *k* (*k* is set to 5 by default) similar proteins in common, i.e., |S(*Q*) ∩ S(*P*)| ≥ *k*, protein *P* is regarded as a *template protein* for protein *Q*. Subsequently, a template protein set for protein *Q*, denoted as *TPS*, is obtained that consists of both glycoproteins and non-glycoproteins. For protein *Q*, voting score *V*_*G*_ is calculated as Σ_*i*_|S(*Q*) ∩ S(*P*_*i*_)|^2^, where each *P*_*i*_ is a glycoprotein and *P*_*i*_ ∈ *TPS*; similarly, voting score *V*_*NG*_ is calculated as Σ_*j*_|S(*Q*) ∩ S(*P*_*j*_)|^2^, where each *P*_*j*_ is a non-glycoprotein and *P*_*j*_ ∈ *TPS*. The score of the first stage of N-GlyDE for protein *Q* is defined as *V*_*G*_/(*V*_*G*_ + *V*_*NG*_) and is further used for integration.

### Second stage of N-GlyDE

The second stage of N-GlyDE uses SVM to predict for each N-X-S/T sequon whether the asparagine in the sequon is glycosylated. An *l*-mer of the protein sequence with the asparagine located at the center is used to derive its features. To determine *l*, we conducted a preliminary study on the second-stage dataset using only gapped dipeptides as features with *l* ranging from 21 to 29. We selected *l* = 25 for our prediction based on the best MCC as shown in Supplementary File 2: Table S3. For the asparagine (in N-X-S/T sequon) within half-length of either terminus, the dummy amino acid ‘X’ was used to fill up the empty positions. As described below, all of the 3080 sequon-containing *l*-mers in the second-stage dataset used gapped dipeptides, surface accessibility, and secondary structure as input features.

### Gapped dipeptide features

**Figure 5:**
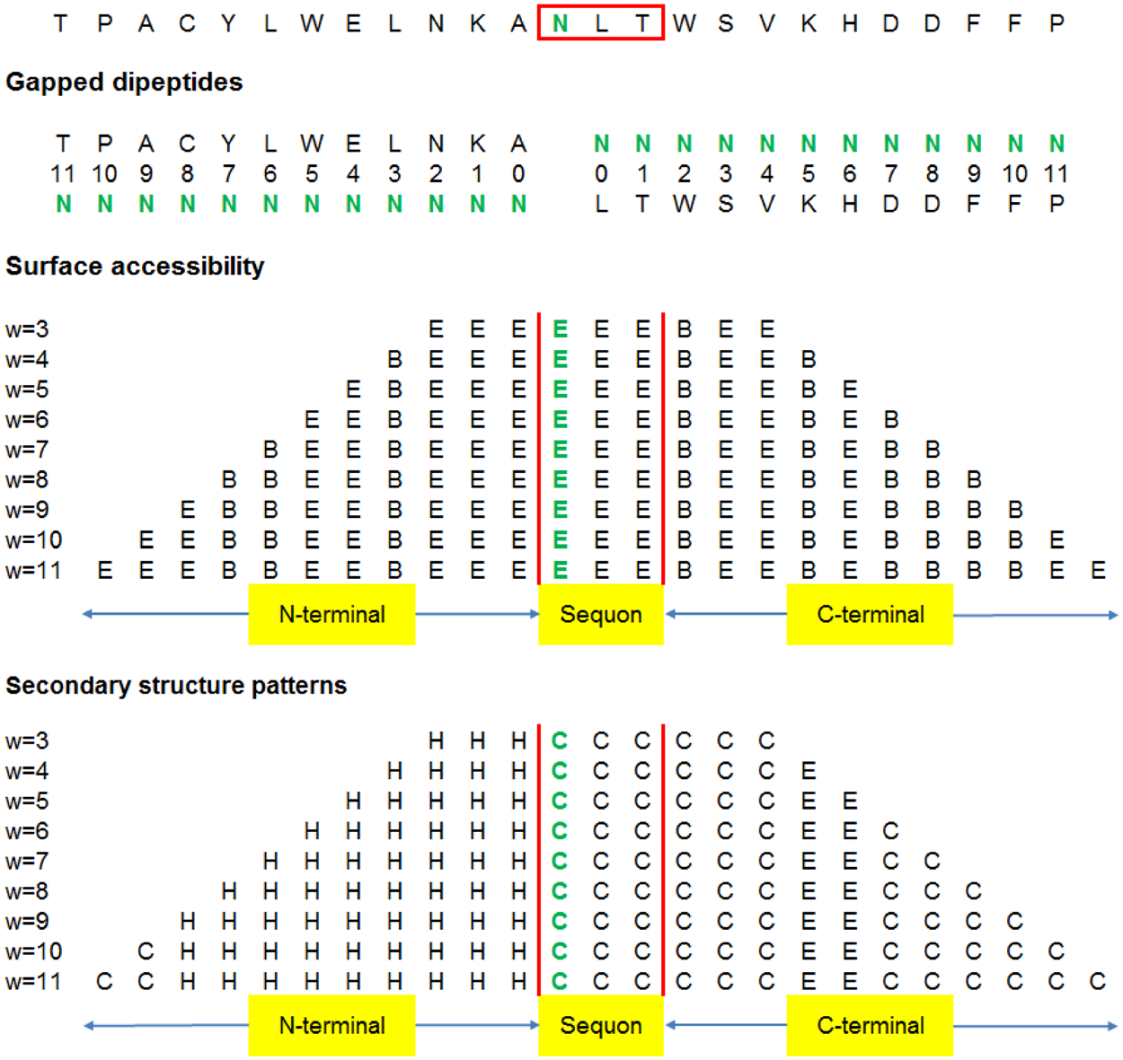
Illustrations of gapped dipeptides and sequence windows with lengths 3 ≤ *w* ≤ 11 used to derive secondary structure and surface accessibility features.

As asparagine of the sequon is at the center of a 25-mer sequence, we consider gapped dipeptides *AkN* and *NkA* of both directions, where *A* represents an amino acid, having a gap of *k* (0 ≦ *k* ≦ 11) from the asparagine on the N- and C- termini, respectively, as shown in Fig. 5. Both N1T and N1S are ignored because they lack the power to discriminate between glycosylated sequons and non-glycosylated sequons. This yields 23 gapped dipeptides from each *l*-mer. From the entire 3080 *l*-mers in the dataset, we obtain 460 gapped dipeptides and count the number of occurrences of each gapped dipeptide in glycosites and non-glycosites. Then we define the gapped dipeptide ratio for each gapped dipeptide as the odds ratio calculated by the percentage of its occurrences in glycosites divided by the percentage of its occurrences in non-glycosites. Normalization of GDRs for a specific gap *k* in the entire 3080 *l*-mers is calculated as (GDR – the minimum GDR) divided by (the maximum GDR – the minimum GDR). Thus, for each 25-mer, the normalized GDR ranging from 0 to 1 is encoded as an input feature in SVM for N-linked glycosite prediction.

### Pattern-based surface accessibility (SA) and secondary structure (SS) features

Secondary structure is used as a feature, since it is reported that alpha helices tend to rarely N-linked glycosylate than coils and sheets^32^. Surface accessibility is employed as another feature because N-linked glycosylation tends to occur at extracellular regions of proteins with the side chain of asparagine in the sequon exposed to the surface. Given a protein sequence, NetSurfP (ver. 1.0)^35^ is used to predict surface accessibility, E (exposed) or B (buried), and secondary structure, H (helix), E (beta sheet) or C (coil), for each amino acid in the protein. Each sequon-containing *l*-mer is represented by the corresponding predicted surface accessibility and secondary structure, respectively. Instead of directly using the predicted SA and SS as features, we propose using a pattern-based approach to find significant patterns to encode these two types of features for dimension reduction as described below.

To generate patterns, each sequon-containing *l*-mer is divided into three regions: N-terminal, sequon, and C-terminal regions. For the sequon, a sequence window of fixed length three is used to obtain the predicted SA and SS patterns. A sequence window of length *w*, 3 ≦ *w* ≦ 11, is used to partition the predicted SA and SS in the corresponding N-terminal (starting from positions 1–10 and ending at position 12 for *l* = 25) and C-terminal regions (starting from position 16 and ending at positions 18–25 for *l* = 25), respectively, into patterns of length *w*, as shown in Fig. 5. Patterns with occurrences below 1% of the 3080 *l*-mers are considered less prominent patterns and grouped together as a single pattern. To identify useful patterns of appropriate length on N-terminal and C-terminal regions, we calculate the *average pattern deviation* of all patterns with length *w*, denoted as *APD*_*w*_ as follows. For each pattern *i* of length *w*, let *e*_*i*_ and *n*_*i*_ denote the number of occurrences of the pattern in glycosites and non-glycosites, respectively; a background cut-off threshold (*z*) is determined as the number of glycosites divided by the number of non-glycosites. Then *APD*_*w*_ is defined as

1 $$AP{D}_{w}=\frac{\sum _{i}|[({e}_{i}/{n}_{i})-z]|\times ({e}_{i}+{n}_{i})}{\sum _{i}({e}_{i}+{n}_{i})}$$

Among *APD*_*w*_ of various pattern length *w*, the length w* yielding the maximum *APD*_*w*_ is selected to encode the corresponding SA and SS features for N-terminal and C-terminal regions. In the case of surface accessibility, a pattern length of six yields the highest *APD*_*w*_ for both N- and C-terminal regions; all patterns of length six are used to encode the feature. For the secondary structure feature, pattern lengths of six and nine produce the maximum *APD*_*w*_ for N-terminal and C-terminal regions, respectively. For each *l*-mer, SA and SS features for the three regions are encoded in the following order: N-terminal, sequon, and C-terminal regions. Surface accessibility results in 79 features, i.e., 36, 8, and 35 patterns for N-terminal, sequon, and C-terminal regions, respectively. Likewise, secondary structure yields 54 features, i.e., 18, 12, and 24 patterns for N-terminal, sequon, and C-terminal regions, respectively. The SA and SS features are encoded by two binary vectors of length 79 and 54, respectively, where the value of ‘1’ indicates a matched pattern and ‘0’ indicates an unmatched pattern. Search space is dramatically reduced when using pattern-based encoding rather than conventional feature encoding.

### SVM model training

We use the LIBSVM^36^ package to train our SVM models. LIBSVM provides kernel options such as polynomial, Gaussian radial basis function (RBF), and sigmoid. Among these, we choose the most commonly used RBF as the kernel function. Furthermore, according to a study comparing kernel functions^37^, the RBF kernel with an appropriate regularization (i.e., the penalty parameter *C* in the RBF kernel) determined during training yields an optimal predictor that minimizes the approximation errors of the classifier. For each sequon, the input features for training include 23 features from gapped dipeptides, 79 SA patterns encoded as a binary vector, and 54 SS patterns encoded as a binary vector, a total of 156 features. Ten-fold cross validation is used to optimize the parameters in the RBF kernel function using a grid search. Parameters *C*, ranging from 2^−5^, 2^−3^, …, 2^15^, and the kernel width parameter (*γ*) ranging from 2^−15^, 2^−13^, …, 2^3^, are optimized and selected from training based on the highest accuracy achieved in the grid search. With the optimized *C* and *γ* parameters, the corresponding SVM model is used to predict the test set and report a glycosite probability score. The score threshold to determine a positive prediction, chosen from a range of 0.25 to 0.75, is selected based on the maximum MCC obtained from each training set. The prediction performance of each test fold is estimated based on the corresponding *C*, *γ*, and score thresholds from the training folds. The model that yields the highest MCC on the test fold is used to predict the independent dataset.

### Integration of two stages for final prediction

To integrate the two stages of N-GlyDE, the prediction score of the first stage is used as a measure to adjust the prediction score of the second stage. We consider two prediction score thresholds of the first-stage prediction for weight adjustment. Specifically, if the prediction score of the first stage for the query protein is below 0.4, the prediction scores of the second stage for all the sequons in the protein are reduced by 20%. If the prediction scores of the first stage for the query protein are above 0.8, the prediction scores for all the sequons of the second stage are increased by 10%. Otherwise, the prediction scores for the sequons of the second stage remained unchanged. If a sequon has a final prediction score above 0.6, the sequon is predicted as a glycosylation site.

### Performance evaluation measures

For performance comparison, we evaluated the prediction results of the N-X-S/T sequons on accuracy, precision, sensitivity, specificity and MCC, defined as

2 $$Accuracy=\frac{TP+TN}{TP+TN+FP+FN}$$

3 $$Precision=\frac{TP}{TP+FP}$$

4 $$Sensitivity=\frac{TP}{TP+FN}$$

5 $$Specificity=\frac{TN}{TN+FP}$$

6 $$MCC=\frac{TP\times TN-FP\times FN}{\sqrt{(TP+FP)(TP+FN)(TN+FP)(TN+FN)}}$$

Furthermore, the receiver operating characteristic curve was constructed by selecting 1000 cut-off values between 0 and 1; the area under the curve was also reported to evaluate the performance.

## Supplementary information

Dataset 1, Dataset 2, Dataset 3

Supplementary Figures and Tables

Source Code of N-GlyDE

## Data Availability

N-GlyDE is available as a web server at http://bioapp.iis.sinica.edu.tw/N-GlyDE/. All datasets involved in this study are available in Supplementary File 1.
