# Supplementary material for: N-GlyDE: a two-stage N-linked glycosylation site prediction incorporating gapped dipeptides and pattern-based encoding
Source: Sci Rep. 2019 Nov 4;9:15975. doi: 10.1038/s41598-019-52341-z (PMC6828726; doi:10.1038/s41598-019-52341-z)
Supplement: Supplementary file 2 — Supplementary Figures and Tables [file 41598_2019_52341_MOESM2_ESM.pdf]

N-GlyDE: a two-stage N-linked glycosylation site prediction incorporating gapped dipeptides and pattern-based encoding

Thejkiran Pitti[1,2,3], Ching-Tai Chen[1,*], Hsin-Nan Lin[1], Wai-Kok Choong[1], Wen-Lian Hsu[1] and Ting-Yi Sung[1,*]

[1]Institute of Information Science, Academia Sinica, Taipei 11529, Taiwan and [2]Institute of Bioinformatics and Structural Biology, National Tsing Hua University, Hsinchu 30013, Taiwan, [3]Bioinformatics Program, Taiwan International Graduate Program, Institute of Information Science, Academia Sinica, Taipei 11529, Taiwan

*To whom correspondence should be addressed.

Supplementary Figures

1. Figure S1. Scatter plot of N-GlyDE first-stage and final (integrating both stages) prediction scores on (A) glycosylated sequons and (B) non-glycosylated sequons in the independent test dataset.
2. Figure S2. ROC curves and AUCs of the second-stage SVM models derived from different combination of features.

Supplementary Tables

1. Table S1. Prediction results of VEGFR2 (P35968) on each sequon by N-GlyDE.
2. Table S2. N-GlyDE's prediction results of fibronectin (P02751) in the independent data set.
3. Table S3. Benchmark performance of SVM model using only gapped dipeptides feature on different sequence windows.

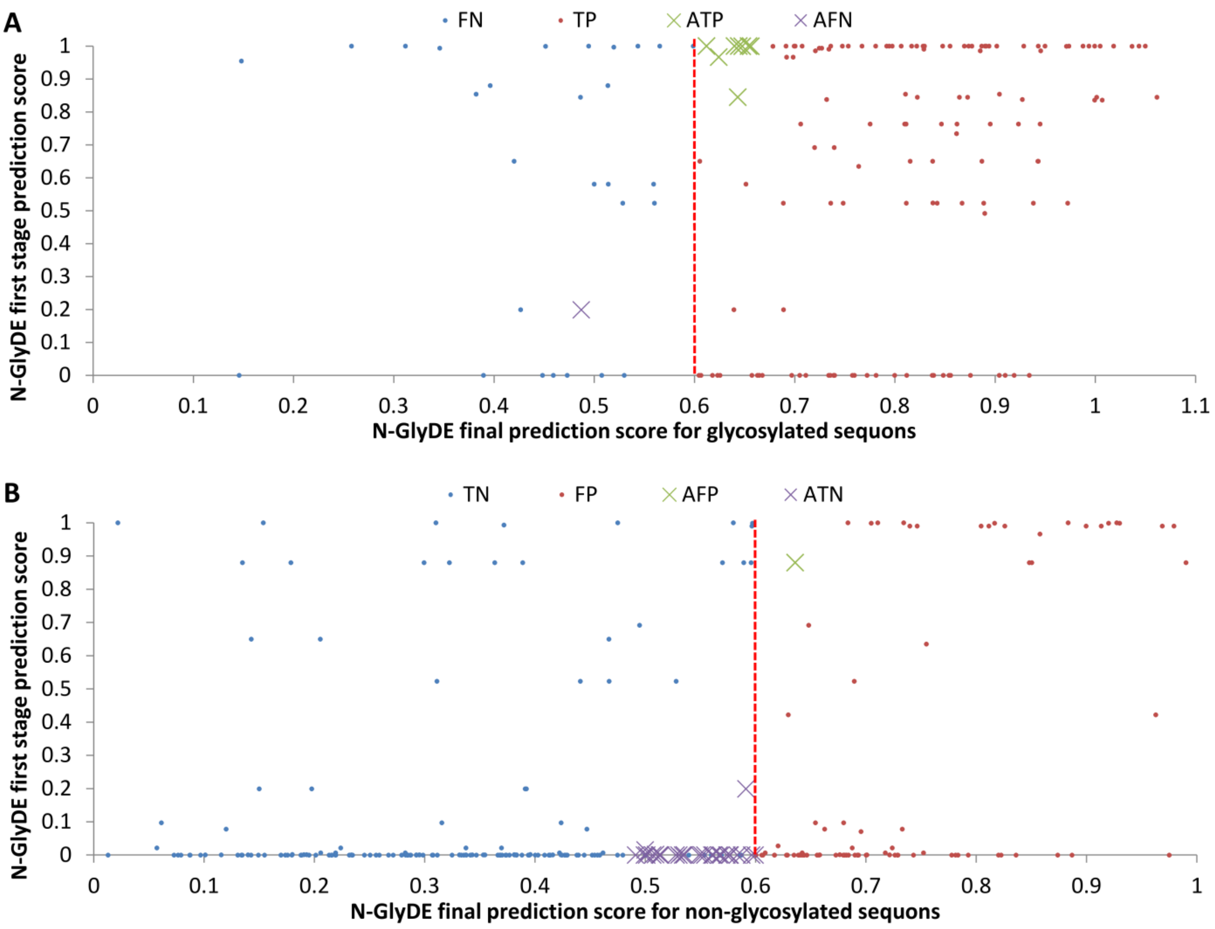

Figure S1. Scatter plot of N-GlyDE first-stage and final (integrating both stages) prediction scores on (A) glycosylated sequons and (B) non-glycosylated sequons in the independent test dataset. Dotted line at 0.6 represents the threshold for the asparagine to be predicted as glycosite. Sequons whose glycosite binary prediction results remain unchanged after integrating the first-stage prediction score are marked as dots. The other sequons are represented with cross that include adjusted true positive (ATP), adjusted true negative (ATN), adjusted false positive (AFP) and adjusted false negative (AFN), respectively.

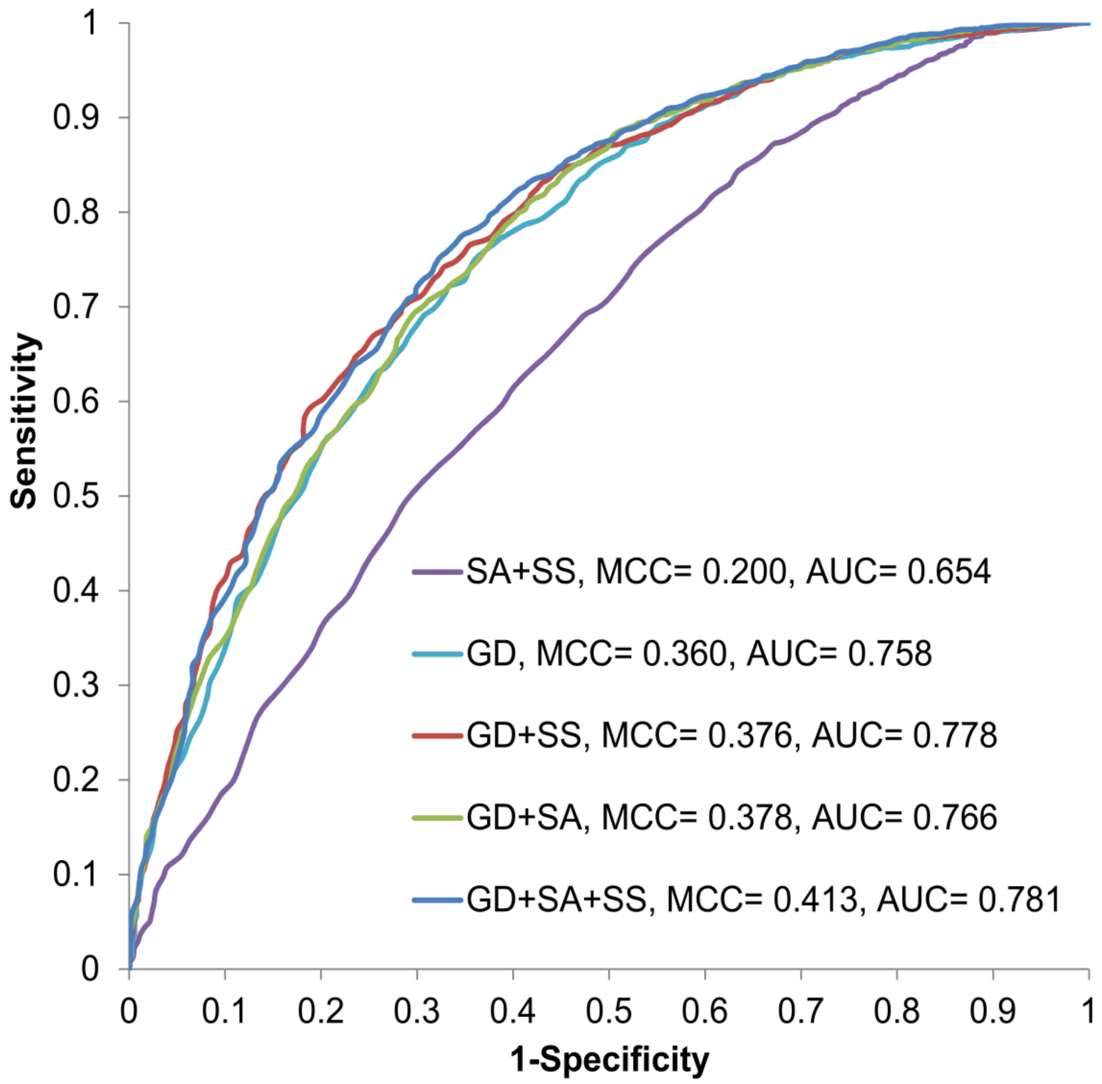

Figure S2. ROC curves and AUCs of the second-stage SVM models derived from different combination of features. The feature sets compared are SA+SS, GD, GD+SS, GD+SA, GD+SA+SS, where GD, SA and SS represent gapped dipeptides, surface accessibility and secondary structure, respectively. The figure shows that GD made the large contribution to AUC.

Table S1. Prediction results of VEGFR2 (P35968) on each sequon by N-GlyDE.

**N-position in sequon**	**UniProt evidence**	**Chandler *et al.'s* results**	**N-GlyDE's prediction score**	**N-GlyDE's predicted glycosites**
46	Sequence annotation	Observed	0.979	Y
66	Sequence annotation	NF*	0.682	Y
96	Sequence annotation	Observed	0.748	Y
143	Exp. validated	Observed	0.885	Y
158	Sequence annotation	Observed	0.794	Y
245	Exp. validated	Observed	0.874	Y
318	Exp. validated	Undetected by mass spectrometry	0.779	Y
374	Sequence annotation	Observed	0.690	Y
395	Sequence annotation	Observed	0.866	Y
511	Sequence annotation	Observed	0.786	Y
523	Sequence annotation	Observed	0.843	Y
580	Sequence annotation	Observed	0.664	Y
613	Sequence annotation	Observed	0.924	Y
619	Sequence annotation	Observed	0.645	Y
631	Sequence annotation	Undetected by mass spectrometry	0.927	Y
675	Sequence annotation	Observed	0.725	Y
704	Sequence annotation	Observed	0.759	Y
721	Sequence annotation	Observed	0.761	Y
923	No annotation	Not studied in Chandler *et al.*'s study	0.681	Y
1256	No annotation	Not studied in Chandler *et al.*'s study	0.475	N
1300	No annotation	Not studied in Chandler *et al.*'s study	0.313	N

NF* represents the only sequon found in human VEGFR2 but not in murine VEGFR2, thus not studied in Chandler *et al.*'s studies. Exp. validated stands for experimentally validated N-glycosites. Y represents the asparagine predicted as N-glycosites and N represents the asparagine predicted as non-glycosites by N-GlyDE.

Table S2. N-GlyDE's prediction results of fibronectin (P02751) in the independent data set.

**N-position in sequon**	**UniProt evidence**	**N-GlyDE's prediction score**	**N-GlyDE's predicted glycosites**	**Positive-oriented gapped dipeptides**	**Negative-oriented gapped dipeptides**
430	Exp. validated	0.838	Y	**N**0Y	--
528	Exp. validated	0.420	N	--	**N**4K
542	Exp. validated	0.943	Y	H6**N**, L0**N**	--
877	Exp. validated	0.887	Y	Y0**N**	--
1007	Exp. validated	0.943	Y	--	--
1236	No annotation	0.205	N	--	**N**2P
1244	Exp. validated	0.815	Y	Y0**N**	--
1417	No annotation	0.143	N	--	**N**2P
1813	No annotation	0.467	N	--	**N**2P
2108	Exp. validated	0.605	Y	H6**N**	--

Exp. validated stands for experimentally validated N-glycosites. Y represents the asparagine predicted as N-glycosites and N represents the asparagine predicted as non-glycosites by N-GlyDE.

Table S3. Benchmark performance of SVM model using only gapped dipeptides feature on different sequence windows.

***l*-mer**	**Accuracy**	**Precision**	**Sensitivity**	**Specificity**	**MCC**
21	0.731	0.796	0.801	0.591	0.394
23	0.737	0.799	0.807	0.596	0.405
25	0.733	0.819	0.781	0.638	0.432
27	0.739	0.816	0.785	0.648	0.426
29	0.743	0.809	0.803	0.622	0.424
